# Urinary Claudin-2 Measurements as a Predictor of Necrotizing Enterocolitis: A Pilot Study

**Published:** 2015-10-01

**Authors:** Brian P Blackwood M.D., Douglas R Wood B.S., Carrie Y Yuan B.S., Joseph D Nicolas, Anne Griffiths M.D., Karen Mestan M.D., Catherine J Hunter M.D.

**Affiliations:** 1 Ann and Robert H. Lurie Children’s Hospital of Chicago, Dept. of Pediatric Surgery 225 E. Chicago Ave, Box 63 Chicago, IL 60611; 2 Rush University Medical Center, Dept. of General Surgery 1750 W. Harrison Street, Suite 785 Chicago , IL 60612; 3 Northwestern University, Feinberg School of Medicine, Dept. of Pediatrics 225 E. Chicago Ave, Box 63 Chicago, IL 60611

**Keywords:** NEC, Neonate, Claudin, Biomarker, Diagnosis

## Abstract

Background: Necrotizing Enterocolitis (NEC) affects 5-10% of NICU patients where initially patients may have only nonspecific clinical findings. A noninvasive tool for detection would aid in diagnosis. Increased urinary claudins have been associated with active adult inflammatory bowel disease.

Methods: Institutional Review Board approval was obtained. Neonatal intestinal tissue samples were obtained from patients with and without NEC. Immunofluorescence analysis of claudin-2 was performed on the intestinal tissue. Thirty two urine samples were collected from 6 NICU patients. Proteins were extracted and urinary claudin-2 expression was measured using Western Blot Analysis. All sample concentrations were normalized to urinary creatinine. Differences were analyzed with ANOVA or Student’s T-test. Findings were correlated to the patient’s clinical status.

Results: Neonatal intestinal immunofluorescence analysis revealed that claudin-2 is present in healthy intestinal epithelium and is decreased in NEC intestinal tissue (p=0.0001). Of the six patients evaluated, three patients had NEC. All 3 patients with NEC had spikes in urinary claudin-2 levels (p<0.001, p<0.001, p 0.0598 respectively). Spikes did not appear to correlate with other etiologies of neonatal sepsis, medication use or need for mechanical ventilation. Levels during active NEC were almost twice that of NEC-free periods (p<0.0001).

Conclusion: A tool for early detection would facilitate early intervention and potential prevention of severe NEC. Preliminary findings indicate that urinary claudin-2 may represent a potential biomarker for NEC worth further investigation.

## INTRODUCTION

Diagnosis of NEC can be difficult, and identifying patients at the onset of disease remains a challenge. Bell’s Criteria has been the mainstay for the diagnosis and staging of NEC for the last three decades [1]. While this staging system is widely used, the signs and symptoms of early stage NEC are nonspecific. Difficulty with the early diagnosis of NEC has led to the search for a more reliable way to both identify those patients that are at risk for developing NEC and to help aid in early diagnosis. Early markers of NEC have been investigated such as elevated cytokines in extremely low birth weight infants, but this was proven not to be helpful as the cytokines only became elevated after the diagnosis of NEC was already made [2]. There has been interest in identifying biomarkers for NEC, as an example urinary intestinal fatty acid binding protein (I-FABP) has been proposed as a potential marker. Others have suggested that diagnostic algorithms using clinical and laboratory data may be helpful in a more reliable early diagnosis [3-6]. These studies have shown some promise, but have not been validated. 

Tight Junctions are cell to cell adhesion complexes in the apical portion of intestinal epithelial cells [7-8]. There is evidence that these TJ proteins are internalized or degraded in response to stress or injury, like that seen in NEC [9]. Claudins are interesting TJ proteins because they have been identified as potential biomarkers for inflammatory bowel disease in adult patients as well as for intestinal injury in mouse models [10]. Given these current findings, we hypothesize that urinary claudin-2 levels will be elevated in neonates with NEC as compared to those without NEC, and therefore urinary claudin-2 levels could serve as a potential predictor of early NEC.

## MATERIALS AND METHODS

Neonatal Intestinal Samples:

Ann and Robert H. Lurie Children’s Hospital Institutional Review Board approval and written consent from the next of kin, caretakers, or guardians on behalf of the minors/children were obtained. Neonatal intestinal tissue samples were obtained from infants undergoing bowel resection. The type of tissue, reason for surgery, and corrected gestational age were recorded at the time of collection. The intestinal samples were categorized into two different groups: active NEC (intestine removed during surgery for perforation and sepsis) and those without NEC (e.g. ostomy takedown, intestinal atresia). Tissue was collected and preserved in Optimal Cutting Temperature Embedding Medium (OTC, Tissue-Tek) at -80°C. These samples were cut into 4 micrometer sections for immunofluorescence analysis of claudin-2 as described later.

Immunofluorescence of Intestinal Tissue Samples:

Tissue samples of neonatal intestine preserved in OCT were cut into 4 micrometer sections. The tissue was washed with phosphate buffered saline (PBS), and then fixed with 1 % paraformaldehyde. The cryo-sections were then blocked with PBS/Triton and 10% normal goat serum, and then incubated in the primary antibody for claudin-2 (Invitrogen). The primary antibody was removed prior to incubation in the secondary antibody, Alexa Fluor® 594 Goat Anti-Mouse IgG Antibody (Invitrogen). The membrane was mounted with Fluoroshield with DAPI (Sigma, F6057) and examined with our Nikon A1R confocal microscope.

Urine sample collection and treatment:

A total of 32 urine samples were collected by placing a sterile cotton ball into the diapers of the 6 neonatal patients that were high risk for developing NEC. Three of the patients went on to develop NEC during the study period. The urine was then squeezed from the cotton ball into a 3ml collection tube. The urine was then put on ice immediately and then frozen in a -80°C freezer for storage. 

Urinary creatinine was measured by ELISA. After thawing the specimens were centrifuged at 1500g for 15 minutes at 4°C. A 40ul sample of supernatant was mixed with 160 µl of High Performance Liquid Chromatography (HPLC) grade ultrapure water for a 1:5 dilution. Additional dilutions (1:10, 1:20, and 1:40) were performed. Samples were kept on ice until the urinary creatinine concentration was determined.

Protein was extracted from each urine sample for Western Blot analysis. The samples were removed from the -80°C freezer and thawed on ice. Once thawed, the samples were centrifuged at 1500g for 15 minutes at 4°C. 100 µl of the supernatant was then combined with 35ul of 6x Laemmli Sample Buffer (Morganville Scientific) and then boiled for 3 minutes and then cooled. Prepared samples were assayed using by Western Blot Analysis. 

Creatinine Measurement and Use for Sample Normalization:

Urine samples were assayed in triplicate with the Urinary Creatinine Assay Kit (Caymen Chemical Company). A plate reader (Molecular Devices Gemini XS) was used to measure the absorbance of each sample. Using the absorbance value of the standards a standard curve was plotted. Next the creatinine concentration of each sample was found using the equation Creatinine (mg/dl) = [sample absorbance – (y-intercept)/slope] x sample dilution. These creatinine concentrations were then used to normalize our Western Blot urinary claudin-2 concentrations.

Claudin-2 Western Blot Analysis:

Western Blot analysis measured the protein expression of claudin-2 in the urine samples. The samples were vortexed and 15 µl were electrophoresed in 15% SDS-PAGE and then electotransferred onto Nitrocellulose membranes (Bio-Rad). The membrane was then blocked in 5% Blotting Grade Blocker (Bio-Rad) and PBS with 0.05% Tween for 2 hours. The membranes were incubated overnight with Mouse Anti-claudin-2 (Invitrogen) at a concentration of 1 to 500, at 4°C. And thereafter, washed three times with PBS/ Tween before the secondary antibody, Anti Mouse (Invitrogen), was added for 2 hour incubation at room temperature. After washing and the membrane was developed in Western Blotting Detection Reagent (Amersham) for 5 minutes before being transferred to film. Band densities were measured using Image Lab Software (Bio-Rad) and normalized to urinary creatinine concentration. 

Statistical Analysis:

Graphs were generated using Excel, and statistical analysis (ANOVA or Student’s T-test) was performed using GraphPad Prism 6. Differences were considered significant at p<0.05.

## RESULTS

Intestinal claudin-2:

Staining confirmed that claudin-2 was present in the intestinal epithelium of normal neonates. When we compared those tissues of neonates with NEC versus those without NEC we found that there was a reduction in the amount of claudin-2 present in those samples from patients with NEC versus those patients without NEC (Fig.1). Mean Fluorescence Intensity reveals that this is a significant decrease in claudin-2 (p=0.0001) (Fig.1). 

**Figure:**
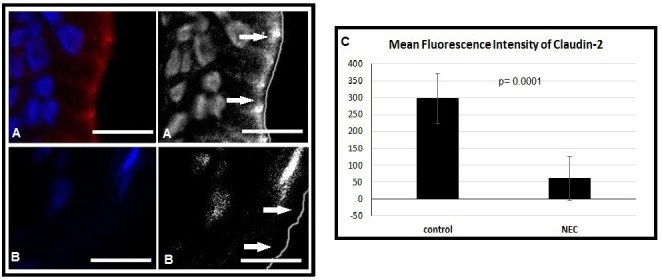
Figure 1: A) Claudin-2 is identified in the intestinal epithelial cells of a control infant that does not have NEC, indicated by the red stain and the white arrows. B) Claudin-2 is missing from the intestinal epithelial cells of an infant that does have NEC, indicated by the absence of red staining and the white arrows. C) MFI reveals a significant decrease in the amount of claudin-2 in the intestinal epithelial cells of the NEC infant versus the control infant (p=0.0001).

Urinary Claudin-2:

To evaluate urinary claudin-2 as a potential marker for NEC, we analyzed the amount of claudin-2 protein in the urine of neonatal patients by Western blot. Urinary samples were obtained from a total of six patients. Three of these patients developed NEC during the collection time course and three did not. All three patients who developed NEC were found to have spikes in urinary claudin-2 levels during their NICU admission, two of which were shown to be significantly elevated (p<0.001, p<0.001, p= 0.0598 respectively). Additionally, two of the three patients’ spikes in urinary claudin-2 directly coincided with their having active NEC (Fig.2). These spikes in urinary claudin-2 did not appear to correlate with other etiologies of neonatal sepsis, medication use, or need for mechanical ventilation. Further analysis of the patients with NEC showed that urine claudin-2 levels during periods of active NEC were almost twice that of the levels during NEC-free periods (p<0.0001) (Fig.3). Additionally, these spikes in urine claudin-2 during NEC periods were also significantly higher than the claudin-2 levels in the control neonates (P=0.0506) (Fig.3). Of note, the control neonates did not develop NEC at any time during their clinical course.

**Figure:**
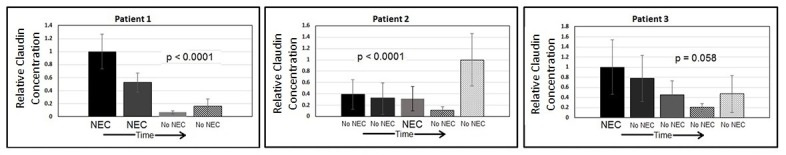
Figure 2: All three patients with diagnosed NEC had spikes in their urinary claudi-2 levels during their NICU admission. Patient 1 and Patient 3 had spikes that directly coincided with their having active NEC.

**Figure:**
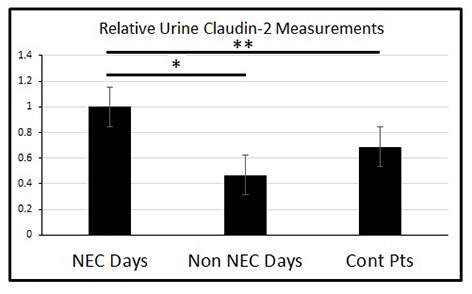
Figure 3: Patients had nearly double the measurement of urinary claudin-2 during periods of active NEC vs. NEC free periods (*p<0.0001). The elevated urinary claudin-2 levels were also significantly higher than the control patients (**p=0.0506).

## DISCUSSION

Claudin-2 is a specific TJ protein in the formation of channels that are selective for the movement of cations and water from one cell to another [11, 12]. Claudin-2 is also a TJ protein in the ileum of both humans and mice, making it a great candidate for experimental study [13-15]. Given that TJ protein degradation is implicated as an early event in NEC and that urinary claudin levels have been identified as a potential marker for this breakdown, we hypothesized that urinary claudin-2 levels would be elevated in neonates with NEC as compared to those without NEC, and therefore urinary claudin-2 levels could serve as a potential predictor of early NEC. In our immunofluorescence analysis of the neonatal intestinal tissue we not only showed claudin-2 to be present in the intestinal epithelium, but we also showed that intestinal segments take from infants with advanced surgical NEC had a reduced protein expression of claudin-2. Furthermore, we showed that urinary cluadin-2 levels were elevated in neonates that had NEC versus those without NEC. Of the three patients that had NEC, two had significant increases in urinary claudin-2 levels (p<0.001). Also in two of these three patients, the spikes in urinary claudin-2 directly coincided with periods of active NEC, and even more impressively the urinary claudin-2 was almost doubled when looking at periods of active NEC versus NEC-free periods (p<0.0001). The non-NEC control patients never developed NEC during their hospital course and were not identified as having sepsis from any other etiology during this study period.

A limitation of our study is the number of patients that we enrolled in the study for urine collection. We designed this project as a pilot study in preparation for a larger prospective cohort study. While we did have a total of 32 urine samples, these samples were obtained from a total of only 6 patients (3 patients who developed NEC and 3 patients without NEC). Analysis of additional urine samples from more patients would give more power to the study and validate our initial findings. Additionally, the claudin-2 that we are measuring in the urine could be coming from a source other than the diseased bowel, such as the kidney itself. Although it is technically feasible to measure claudin-2 in stool samples, infants with abdominal sepsis tend to have a reduced stool output which would make collection during the early acute disease period unreliable. Collection and analysis of serum could represent another strategy; however infants in the NICU undergo multiple blood draws which may impact their totally circulatory volume. Therefore, our goal was to find more non-invasive strategy to assess the changes in this particular biomarker.

## Conclusion

In conclusion, a tool for early detection would be a valuable asset and facilitate early intervention and potential prevention of severe NEC. Preliminary findings indicate that urinary claudin-2 may represent a potential biomarker for NEC worth further investigation. 

## Footnotes

**Source of Support:** Grant Support from AGA and Children’s Research Foundation

**Conflict of Interest:** None
